# BMP-6 promotes E-cadherin expression through repressing δEF1 in breast cancer cells

**DOI:** 10.1186/1471-2407-7-211

**Published:** 2007-11-13

**Authors:** Shuang Yang, Jun Du, Zhaoqi Wang, Wei Yuan, Yuhuan Qiao, Ming Zhang, Jie Zhang, Songyuan Gao, Jian Yin, Baocun Sun, Tianhui Zhu

**Affiliations:** 1 Medical College of Nankai University, Tianjin, China; 2 Tianjin Medical University Cancer Institute and Hospital, Tianjin, China

## Abstract

**Background:**

Bone morphogenetic protein-6 (BMP-6) is critically involved in many developmental processes. Recent studies indicate that BMP-6 is closely related to tumor differentiation and metastasis.

**Methods:**

Quantitative RT-PCR was used to determine the expression of BMP-6, E-cadherin, and δEF1 at the mRNA level in MCF-7 and MDA-MB-231 breast cancer cells, as well as in 16 breast cancer specimens. Immunoblot analysis was used to measure the expression of δEF1 at the protein level in δEF1-overexpressing and δEF1-interfered MDA-MB-231 cells. Luciferase assay was used to determine the rhBMP-6 or δEF1 driven transcriptional activity of the E-cadherin promoter in MDA-MB-231 cells. Quantitative CHIP assay was used to detect the direct association of δEF1 with the E-cadherin proximal promoter in MDA-MB-231 cells.

**Results:**

MCF-7 breast cancer cells, an ER^+ ^cell line that expressed high levels of BMP-6 and E-cadherin exhibited very low levels of δEF1 transcript. In contrast, MDA-MB-231 cells, an ER^- ^cell line had significantly reduced BMP-6 and E-cadherin mRNA levels, suggesting an inverse correlation between BMP-6/E-cadherin and δEF1. To determine if the same relationship exists in human tumors, we examined tissue samples of breast cancer from human subjects. In 16 breast cancer specimens, the inverse correlation between BMP-6/E-cadherin and δEF1 was observed in both ER^+ ^cases (4 of 8 cases) and ER^- ^cases (7 of 8 cases). Further, we found that BMP-6 inhibited δEF1 transcription, resulting in an up-regulation of E-cadherin mRNA expression. This is consistent with our analysis of the E-cadherin promoter demonstrating that BMP-6 was a potent transcriptional activator. Interestingly, ectopic expression of δEF1 was able to block BMP-6-induced transactivation of E-cadherin, whereas RNA interference-mediated down-regulation of endogenous δEF1 in breast cancer cells abolished E-cadherin transactivation by BMP-6. In addition to down-regulating the expression of δEF1, BMP-6 also physically dislodged δEF1 from E-cadherin promoter to allow the activation of E-cadherin transcription.

**Conclusion:**

We conclude that repression of δEF1 plays a key role in mediating BMP-6-induced transcriptional activation of E-cadherin in breast cancer cells. Consistent with the fact that higher level of δEF1 expression is associated with more invasive phenotype of breast cancer cells, our collective data suggests that δEF1 is likely the switch through which BMP-6 restores E-cadherin-mediated cell-to-cell adhesion and prevents breast cancer metastasis.

## Background

Breast cancer is the most common neoplasm in women. The unique histological features of breast cancer are prominent proliferation of epithelial cells and the formation of ectopic mesenchymal tissue, including cartilage and bone, especially in complex adenomas and benign mixed tumors [1,2]. The association between loss or down-regulation of E-cadherin, an epithelial cell-cell adhesion protein, and progression of breast cancer has been extensively documented [3,4]. Tumor cells acquire invasive properties when E-cadherin-mediated adhesion is inhibited [5,6]. In line with these findings, ectopic expression of E-cadherin in a transgenic mouse model prevented tumor cell invasion and metastasis [7]. Several epigenetic mechanisms are implicated in E-cadherin loss during breast cancer, including hypermethylation of the E-cadherin promoter region at CpG islands [8] and transrepression by specific transcriptional factors. Several zinc finger transcription factors, such as Twist [9,10], Snail1 [11-14], Snail2 [15,16], SIP1 [17], and E12/E47 [16], have been found to bind to the E-box elements in the proximal E-cadherin promoter and repress its transcription. Moreover, a few factors, including ErbB2 [18], TGF-β [19], and estrogen [20], were reported to regulate E-cadherin expression.

Bone morphogenetic protein-6 (BMP-6), a member of TGF-β superfamily, has been characterized as a multifunctional molecule with a distinct ability to induce ectopic cartilage and bone formation [1,21]. *In vitro*, BMP-6 inhibits cell division, promotes cell differentiation, induces ectopic bone formation, and regulates epithelial-mesenchymal interaction [21-24]. Furthermore, a number of recent studies have shown that BMP-6 expression is associated with progression of tumorigenesis. BMP-6 is detected in several human neoplastic epithelial cells including breast, prostate, salivary, rectal, and thyroid carcinomas, and is speculated to be closely associated with tumor metastasis [21,25-29].

δEF1, a member of the zinc finger-homeodomain family of transcription factors [30,31], was originally identified as a binding protein of the lens-specific δ1-crystalline enhancer in chicken [32]. Studies revealed that δEF1 is a widely expressed transcriptional repressor, working through its zinc finger clusters binding to consensus E-box-like sequences, 5'-CA(G/C)(G/C)TG-3' [33-35]. Several recently reported properties mark δEF1 as a potential regulatory factor in various cellular processes during tumor progression. In lung and breast tumor cells, δEF1 has been implicated in epithelial to mesenchymal transition (EMT) [36-38], a process associated with tumor metastasis [39]. Moreover, δEF1 can itself be regulated by hormones in target tissues [40-42]. In one example, δEF1 is up-regulated by estrogen in the chick oviduct and is responsible for estrogen-mediated regulation of the ovalbumin gene [40,41,43]. In human T47D breast carcinoma cells, δEF1 is up-regulated by progesterone via both isoforms of the progesterone receptor (PR) [42].

In this communication, we report that BMP-6 up-regulates the expression of E-cadherin at the mRNA level in breast cancer cells, determined by quantitative RT-PCR and luciferase assay. We demonstrate that this effect is a direct result of BMP-6-indcued down-regulation of δEF1. The inverse relationship between BMP-6/E-cadherin and δEF1 were validated in 16 breast cancer specimens using quantitative RT-PCR. This finding contributes significantly to our understanding of the potential role of BMP-6 and δEF1 in breast tumorigenesis and metastasis.

## Methods

### Tumor Samples

Fresh breast cancer tissues of invasive ductal carcinoma (stage II) were obtained from the Tissue Banking Facility Jointly Supported by TMUCIH (Tianjin Medical University Cancer Institute and Hospital) & NFCR (National Foundation for Cancer Research). The pathological stage and nodal status were obtained from the primary pathology reports. The patients (8 ER positive and 8 ER negative) had a mean age of 52.8 ± 12.1 years and were recruited in the same department. This study was approved by the institutional ethics committee.

### Cell Culture

MCF-7 and MDA-MB-231 cells were maintained in DMEM-high glucose medium (GIBCO BRL, Grand Island, NY, USA) supplemented with 10% FBS (Hyclone, Logan, Utah, USA), penicillin, and streptomycin according to the recommendations of the American Type Culture Collection (ATCC). MDA-MB-231 cells were plated at a density of 2 × 10^4 ^cells/well in 24-well plates and 8 × 10^4 ^in 6-well plates for use in quantitative RT-PCR and luciferase assays, respectively. The cells were cultured in the presence or absence of 200 ng/ml rhBMP-6 (R&D Systems, Minneapolis, MN, USA) in DMEM supplemented with 5% FBS.

### Plasmid Constructs

cDNA fragment encoding the full-length δEF1 sequence was prepared by PCR using the forward primer, 5'-CGCGGATCCAGGATCATGGCGGATGGC-3', and reverse primer, 5'-GAACCTCGAGCGAGCTTCATTTGTCTTCTCTTC-3'. The PCR products were digested with BamHI/XhoI, and cloned into pcDNA6B (Life Technologies, Grand Island, NY, USA). The E-cadherin promoter sequence (-308/+21) was obtained by PCR from human blood genomic DNA and cloned into the pGL4.10 vector (Promega, Madison, WI, USA) as described by Comijn et al. [17]. Mutagenesis of the δEF1 sites in the human E-cadherin promoter was performed using the QuickChange Site-Directed Mutagenesis Kit (Stratagene, La Jolla, CA, USA) with the forward primer E2 box 1: 5'-gctgtggccggCAG**A**TGaaccctcag-3' and reverse primer E2 box 1: 5'-ctgagggttCA**T**CT Gccggccacagc-3'; forward primer E2 box 3: 5'-gctccgggctCA**T**CTGgctgcag c-3' and reverse primer E2 box 3: 5'-gctgcagcCAG**A**TGagccccggagc-3', as described by Comijn et al. [17].

### RNA Extraction and Quantitative RT-PCR

Total RNA was extracted from MCF-7 and MDA-MB-231 cells treated with or without 200 ng/ml rhBMP-6 for various times or from 16 breast cancer specimens using the TRIzol Reagent (Life Technologies, Grand Island, NY, USA). Total RNA (0.5 μg) from each sample was used for first strand cDNA synthesis (M-MLV Reverse Transcriptase, Promega, Madison, WI, USA). Specific products of human δEF1, human E-cadherin, and human BMP-6 were amplified by quantitative PCR using the following primers: δEF1, 5'-GGCCCCAGGTGTAAGCGC-3' (forward), and 5'-CAGGCCCCAGGATTTCTTG C-3' (reverse); E-cadherin, 5'-TGCTGCAGGTCTCCTCTTGG-3' (forward), and 5'-AGT CCCAGGCGTAGACCAAG-3' (reverse); BMP-6, 5'-CAACAGAGTCGTAATCA-3' (forward), and 5'-TTAGTGGCATCCACAAGCTCT-3' (reverse). GAPDH was used as an internal control. Verification of the expression levels of genes was performed by quantitative RT-PCR using EvaGreen (Botium, Hayward, CA, USA). The expression level was expressed as the threshold cycle (C_T_) values of the target and reference gene-GAPDH, which is constitutively expressed and not regulated by treatment with rhBMP-6. Comparison and calculation of C_T _values was used to determine the relative mRNA expression expressed as the fold change of target genes relative to the reference gene.

### Antibodies

The following antibodies (Abs) were used: mouse monoclonal Ab against c-Myc (sc-40, Santa Cruz Biotechnology, CA, USA); goat polyclonal Ab against the N-terminal epitope of δEF1 (ZEB-E20, Santa Cruz Biotechnology, CA, USA); a mouse monoclonal Ab against E-cadherin (610181, BD Transduction Laboratories, KY, USA); and a mouse monoclonal Ab against FLAG-M2 (F-3165, Sigma, MO, USA).

### Western Immunoblot Analysis

MDA-MB-231 cells were solubilized in lysis buffer (50 mM Tris-HCl pH 7.5, 150 mM NaCl, 1% Triton ×-100, 0.5% sodium deoxycholate) supplemented with aprotinin (10 μg/ml), leupeptin (10 μg/ml), and PMSF (1 mM). The lysates were cleared by centrifugation and the protein concentrations were determined using the BCA Protein Assay Kit (Pierce, Rockford, IL, USA). Proteins were separated by SDS-PAGE and transferred onto nitrocellulose membranes (Amersham Biosciences, Piscataway, NJ, USA). Western blotting was performed using standard techniques and immunoreactive bands were detected by chemiluminescence (ECL, Amersham Biosciences, Piscataway, NJ, USA).

### Luciferase Assay

MDA-MB-231 cells were co-transfected with wild-type or mutant human E-cadherin promoter constructs and different amounts of δEF1 expression plasmids (1.5, 3.0, and 6.0 μg/well) in 6-well plates using Lipofectamine 2000 (Invitrogen, Austin, TX, USA). Cells were treated with rhBMP-6 (200 ng/ml) for 24 h after transfection. Lysates were prepared 24 h after treatment. The luciferase activity was then measured using the Dual-Luciferase Reporter Assay System (Promega, Madison, WI, USA) according to the manufacturer's instructions. Luciferase activity was normalized using the Renilla luciferase activity.

### Preparation of small interfering RNAs and Transfection

The target sequence of the siRNA is 5'-TGATCAGCCTCAATCTGCA-3' for human δEF1 as previously reported [36]. The sense and antisense oligonucleotides with the internal loop were synthesized (TaKaRa, Shiga, Japan). These were annealed and ligated into the BamHI and HindIII sites of pSilencer 4.1-CMVneo (Ambion, Carlsbad, CA, USA) to construct the δEF1-specific siRNA expression plasmid according to the manufacturer's instructions. pSilencer 4.1-CMVneo expressing a scrambled siRNA (Ambion, Carlsbad, CA, USA) was used as a control. Transient transfection with siRNAs was performed with Lipofectamine 2000 (Invitrogen, Austin, TX, USA) in MDA-MB-231 cells. G418-resistent clones were isolated over a period of 3–4 weeks. Down-regulation of δEF1 was confirmed by western immunoblot analysis.

### Quantitative CHIP Assays

MDA-MB-231 cells were grown for 24 h up to 80% confluence in the presence or absence of 200 ng/ml rhBMP-6. Cells were cross-linked with 1% formaldehyde and processed using the Chromatin Immunoprecipitation (ChIP) Assay Kit (Updates, Lake Placid, NY, USA). The following antibodies (10 μg) were used: anti-ZEB, a polyclonal antibody raised against N-terminal epitopes of δEF1, or unrelated anti-FLAG control antibody (F-3165, Sigma, MO, USA) with 100 μg of chromatin per CHIP. Purified immunoprecipitated DNA was used for quantitative PCR reactions. The primers used for this analysis were as follows: 5'-AGGCTAGAGGGTCACCGCGTC-3' (forward), and 5'-GCTTTGCAGTTCCGACGCCAC-3' (reverse). Copy numbers for the DNA fragments (-175 to +21) of E-cadherin promoter in each anti-ZEB sample with or without BMP-6 induction were determined and compared to copy numbers of the DNA fragment without IP (input DNA). Anti-FLAG antibody was used as a control for IP reactions. The percentage of the input was then calculated. The final value was the percentage input obtained with specific antibody minus the percentage input obtained with anti-FLAG control antibody. The dissociation curve was determined for each quantitative PCR to ensure that a single band was produced. Each data point represents three independent samples.

## Results

### Expression levels of BMP-6 and E-cadherin are inversely related to that of δEF1 in breast cancer cell lines and in clinical breast cancer specimens

**Figure 1:**
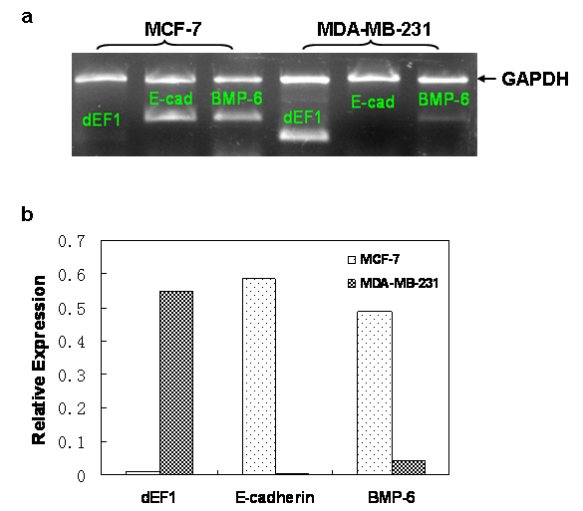
**Expression of BMP-6, E-cadherin, and δEF1 in MCF-7 and MDA-MB-231 breast cancer cells is inversely correlated**. (a) Transcript levels of BMP-6, E-cadherin, and δEF1 in MCF-7 and MDA-MB-231 breast cancer cells were detected by RT-PCR analysis. GAPDH was used as an internal control. (b) The agarose gel electrophoresis data was quantified by UV band scanning. Data represent three independent experiments.

MCF-7 and MDA-MB-231 breast cancer cells feature different capacities for invasiveness and metastasis *in vivo *and, as such, have been intensively used as tumor models during past few years [44]. Our preliminary studies indicated that expressions of BMP-6 and δEF1 were inversely related in MCF-7 and MDA-MB-231 cells. MDA-MB-231 cells, which express high level of δEF1 mRNA, exhibited very low level of BMP-6 (Figure 1). In contrast, MCF-7 cells had markedly reduced δEF1 mRNA levels (Figure 1). Moreover, δEF1 has recently been identified as a direct transcriptional repressor of E-cadherin in these cancer cells [36]. We therefore performed RT-PCR to examine the expression of E-cadherin in MCF-7 and MDA-MB-231 cells. Consistent with the previously reported results [13,36], E-cadherin mRNA expression was high in MCF-7 cells, but undetectable in MDA-MB-231 cells (Figure 1).

**Figure 2:**
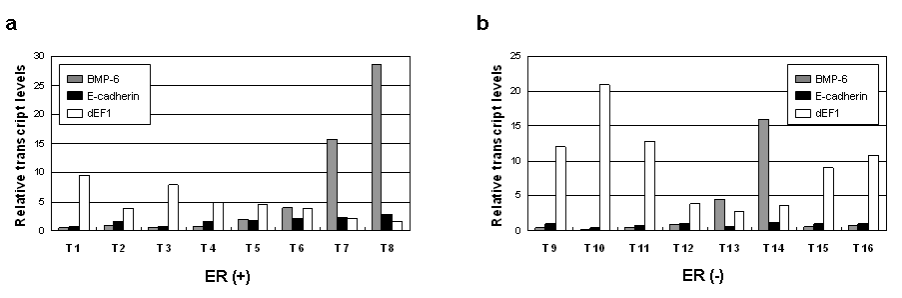
**Relative expression levels of BMP-6, E-cadherin, and δEF1 in 16 breast cancer specimens**. Transcript levels of the three genes were determined in tumor samples from 16 breast cancer specimens by quantitative RT-PCR. GAPDH was used to normalize the individual expression levels.

After establishing the relationship between BMP-6, E-cadherin, and δEF1 in cancer cell lines, we moved on to validate this finding in breast cancer tissue samples. We collected 16 tumor specimens (T1–T8 were ER positive and T9–16 were ER negative) and analyzed their relative mRNA levels using quantitative RT-PCR. As shown in Figure 2b, an inverse correlation between BMP-6/E-cadherin and δEF1 is clearly evident in ER^- ^cancer specimens (7 out of 8: T9, T10, T11, T13, T14, T15, T16). Only in T12 was the low δEF1 level not accompanied by an increased level of E-cadherin/BMP-6. In ER^+ ^cancer specimens, a clear inverse correlation was evident in T1, T3, T7, and T8 (Figure 2a). However, in the other ER^+ ^tumor samples such an inverse correlation was not as evident. In addition, our results revealed that expression of BMP-6 in ER^+ ^breast tumor specimens was comparatively higher than in ER^- ^cases. However, for expression of δEF1 in ER^+ ^and ER^- ^breast tumor specimens, this relationship was reversed; here ER^+ ^tumors had lower δEF1 mRNA levels than ER^-^. Together, these data suggest a strong inverse relationship between the expressions of BMP-6/E-cadherin and δEF1, which could contribute to the invasiveness and metastatic capacity of breast cancer cells.

### BMP-6 down-regulated δEF1 and concurrently promoted E-cadherin transcription in breast cancer cells

**Figure 3:**
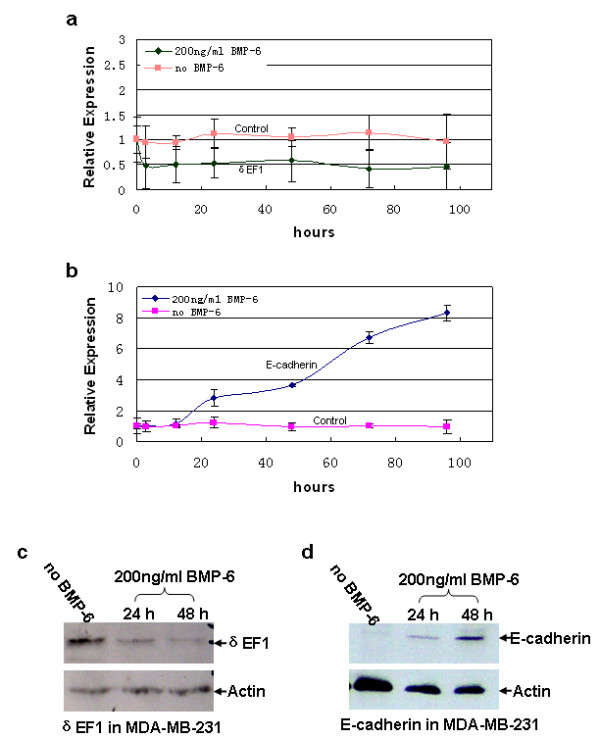
**BMP-6 down-regulates δEF1 and concurrently up-regulates E-cadherin transcription in MDA-MB-231 breast cancer cells**. (a) BMP-6-induced (200 ng/ml) down-regulation of δEF1 mRNA in MDA-MB-231 cells was verified by quantitative RT-PCR. GAPDH was used to normalize the δEF1 level. Data represent three independent experiments. (b) BMP-6-induced (200 ng/ml) up-regulation of E-cadherin mRNA in MDA-MB-231 cells was verified by quantitative RT-PCR. GAPDH was used to normalize the E-cadherin level. Data represent three independent experiments. (c) Western blot with anti-ZEB antibody showing δEF1 expression in MDA-MB-231 cells after culture for 24 or 48 h with BMP-6 (200 ng/ml). Actin expression was used as an internal control. (d) Western blot with anti-E-cadherin antibody showing E-cadherin expression in MDA-MB-231 cells after culture for 24 or 48 h with BMP-6 (200 ng/ml). Actin expression was used as an internal control.

To assess whether a loss of E-cadherin expression in MDA-MB-231 cells is related to the presence of δEF1 suppression as well as a deficiency of BMP-6, the cells were cultured in the presence or absence of rhBMP-6. Total RNA was extracted at 0, 3, 12, 24, 48, 72, and 96 h following BMP-6 treatment. Quantitative RT-PCR analysis demonstrated that BMP-6 treatment for 72 h resulted in an up to 60% decrease in the expression of δEF1 mRNA, compared to the basal level (Figure 3a). Meanwhile, BMP-6 treatment for 96 h increased the expression of E-cadherin more than 8-fold over the level at baseline (Figure 3b). In the sequence of time, the BMP-6-induced down-regulation of δEF1 transcription occurred at as early as 3 h; whereas up-regulation of E-cadherin was observed only after 12 h of BMP-6 induction (Figure 3a and 3b), suggesting that BMP-6-induced down-regulation of δEF1 may well be a pre-requisite to allow the expression of E-cadherin.

To verify these findings, western blot was performed to determine BMP-6-modulated expression of δEF1 and E-cadheirn at the protein level. This time, MDA-MB-231 cells were cultured in the presence or absence of 200 ng/ml rhBMP-6 and total protein lysates were collected at 24 and 48 h following treatment. Western blot analysis revealed that BMP-6 treatment for 48 h significantly reduced the level of δEF1 protein (Figure 3c) with the expression of E-cadherin being up-regulated concurrently (Figure 3d).

### BMP-6-induced transcription of endogenous E-cadherin was suppressed by δEF1 in MDA-MB-231 cells

**Figure 4:**
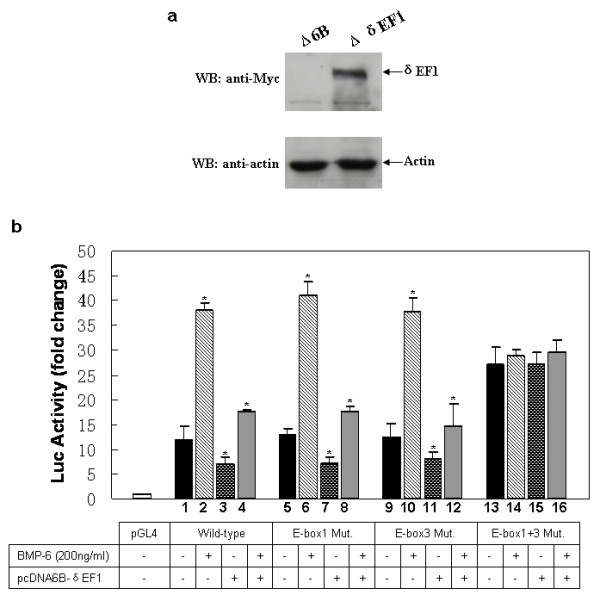
**Ectopic expression of δEF1 is sufficient to attenuate BMP-6-induced transactivation of the E-cadherin in MDA-MB-231 breast cancer cells**. (a) Western blot with anti-myc antibody was performed to show δEF1-myc expression in MDA-MB-231 (Δ6B) and MDA-MB-231 (ΔδEF1) cells. Actin was used as a loading control. (b) MDA-MB-231 cells on a 6-well plate were co-transfected with a δEF1 expression plasmid (2 μg/well) and luciferase E-cadherin promoter constructs (2 μg/well) following treatment with 200 ng/ml rhBMP-6 after 24 h of transfection. The luciferase activity of the extracts was determined 24 h after BMP-6 induction using a Betascope analyzer. Luciferase values are normalized with Renilla activities. * indicates p < 0.05 in unpaired student t test when compared with vector alone. Data represent three independent experiments.

With the knowledge that BMP-6 represses δEF1 transcription and causes up-regulation of E-cadherin in MDA-MB-231 cells, we moved on to examine whether BMP-6 is a *bona fide *stimulator of E-cadherin expression in MDA-MB-231 cells using reporter gene assays. The study showed that BMP-6 significantly stimulated E-cadherin promoter activity of the wild-type -308/+21 reporter gene, compared to the empty-vector transfected control cells (Figure 4b, lane 1 verse 2). Furthermore, we examined whether δEF1 overexpression could repress the BMP-6-induced transcriptional activation of E-cadherin. MDA-MB-231 cells were co-transfected with E-cadherin promoter reporter and δEF1 expression plasmid in the presence of rhBMP-6. Overexpression of δEF1 was confirmed by western blot (Figure 4a). As anticipated, δEF1 expression significantly inhibited the promoter activity of E-cadherin being activated by rhBMP-6 (Figure 4b, lane 2 verse 4), confirming a role for δEF1 in suppressing transcriptional activation of E-cadherin by inducers such as BMP-6.

**Figure 5:**
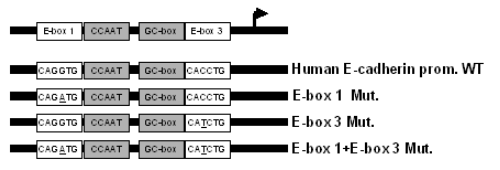
**Scheme of the cloned promoter region of human E-cadherin**. The putative δEF1-binding sites (E-box 1 and E-box 3) and mutations generated in the δEF1-binding sites are indicated.

To further address the specificity of δEF1 in regulation of E-cadherin promoter, we mutated two δEF1-binding sites identified in the human E-cadherin promoter (-308/+21), individually or in combination (Figure 5). The mutant E-cadherin promoter constructs were then co-transfected with δEF1 expression plasmid in the presence of rhBMP-6. A measurement of luciferase activity indicated that δEF1 expression inhibited BMP-6-induced reporter activity of the E-cadherin promoter harboring mutations in either E-box 1 or E-box 3 (Figure 4b, lane 6 verse 8, lane 10 verse 12), an effect similar to that of δEF1 on the wild-type E-cadherin promoter. However, mutations within both E-box elements abolished the repressive effect of δEF1 on the BMP-6-induced reporter gene activity (Figure 4b, lane 13 verse 15, lane 14 verse 16). These data suggested that ectopic expression of δEF1 can attenuate BMP-6-induced transactivation of the E-cadherin promoter through either the E-box 1 or E-box 3 binding sites in MDA-MB-231 cells. Mutation of both E-boxes is required to efficiently interfere with the repressive effect of δEF1 on BMP-6-regulated E-cadherin expression. Moreover, the double mutation of E-boxes not only abolishes the repressive effect of δEF1, it also blunts the induction effect by BMP-6 on E-cadherin promoter. Collectively, we can conclude that in MDA-MB-231 cells, the BMP-6 medicated activation of E-cadherin gene is closely associated with the ability of E-cadherin promoter to bind to its putative suppressors (such as δEF1) through its E-box 1 and E-box 3.

**Figure 6:**
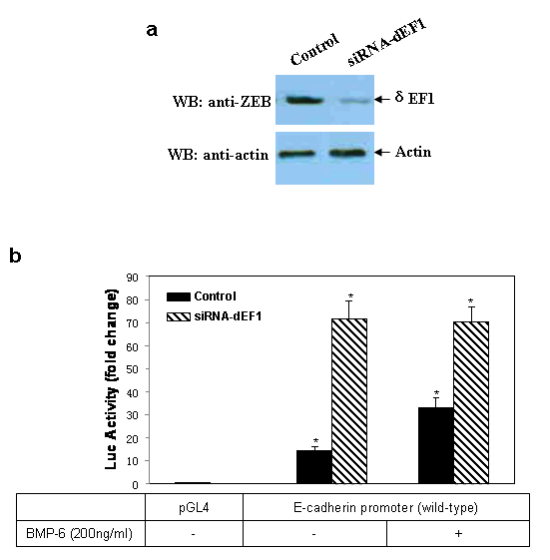
**Repression of endogenous δEF1 abolishes BMP-6-induced transactivation of the E-cadherin promoter in MDA-MB-231 breast cancer cells**. (a) δEF1-specific siRNA plasmid (siRNA-δEF1) was introduced into MDA-MB-231 cells to generate δEF1-interfered stable transfectants. Control cells were treated with a scrambled siRNA. The efficiency of δEF1 protein knockdown was examined by western blot, using an anti-ZEB antibody. Actin was used as a loading control. (b) δEF1-interfered MDA-MB-231 cell were transiently transfected with luciferase E-caherin promoter constructs. After transfection for 24 h, cells were treated with 200 ng/ml rhBMP-6. The luciferase activity of the extracts was determined 24 h after BMP-6 treatment using a Betascope analyzer. Luciferase values are normalized with Renilla activities. * indicates p < 0.05 in unpaired student t test when compared with vector alone.

**Figure 7:**
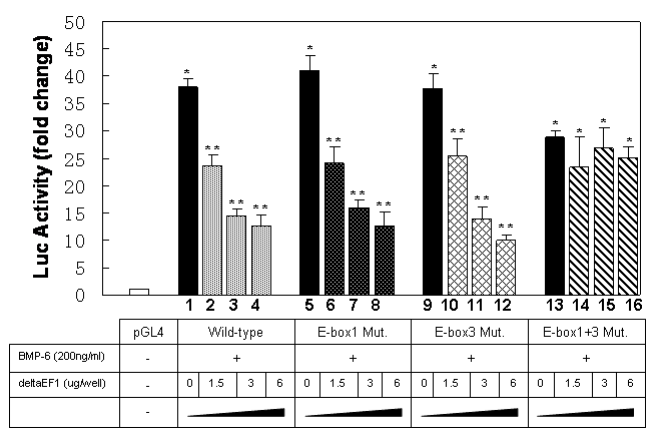
**BMP-6-induced transactivation of E-cadherin is suppressed by δEF1 in a dose-dependent manner**. Wild-type or mutated human E-cadherin promoter constructs were co-transfected with different amounts of δEF1 expression plasmid (1.5, 3.0, 6.0 μg/well) into MDA-MB-231 cells, followed by the treatment with 200 ng/ml rhBMP-6 after 24 h of transfection. The luciferase activity of the extracts was determined 24 h after BMP-6 treatment using a Betascope analyzer. Luciferase values are normalized with Renilla activities. Data represent three independent experiments. * indicates p < 0.05 in unpaired student t test when compared with vector alone. ** indicates p < 0.05 in one-way analysis of variance followed by Dunnett's test when compared with vector alone.

Naturally we were interested in investigating whether knockdown of δEF1 using RNA interference (RNAi) would result in an activation of the promoter. To do so, a small interfering RNA (siRNA) targeting δEF1 or a scrambled control siRNA were stably transfected into MDA-MB-231 cells, followed by treatments with 200 ng/ml rhBMP-6. The knockdown of δEF1 expression was confirmed by western blot (Figure 6a), accompanied by a significant increase in the promoter activity of E-cadherin with or without BMP-6 treatment, compared to the cells transfected with the control siRNA (Figure 6b). The data verified that depletion of δEF1 in MDA-MB-231 cells is sufficient to allow a native expression of the silenced E-cadherin gene. Furthermore, a knockdown of δEF1 abolished the E-cadherin promoter stimulation by rhBMP-6 (Figure 6b), suggesting that the stimulatory effect of BMP-6 on E-cadherin transcription occurs indirectly. Down-regulation of δEF1 is a pre-requisite for BMP-6 to induce the expression of E-cadherin. While the δEF1 suppression of the BMP-6-stimulated E-cadherin promoter activity was dose-dependent, double mutation of E-boxes causes a total abolishment of the δEF1 effect, given that the molecule was unable to exhibit a repression at any dose when both E-boxes were mutated (Figure 7).

### BMP-6 attenuates the association of endogenous δEF1 with E-cadherin promoter

**Figure 8:**
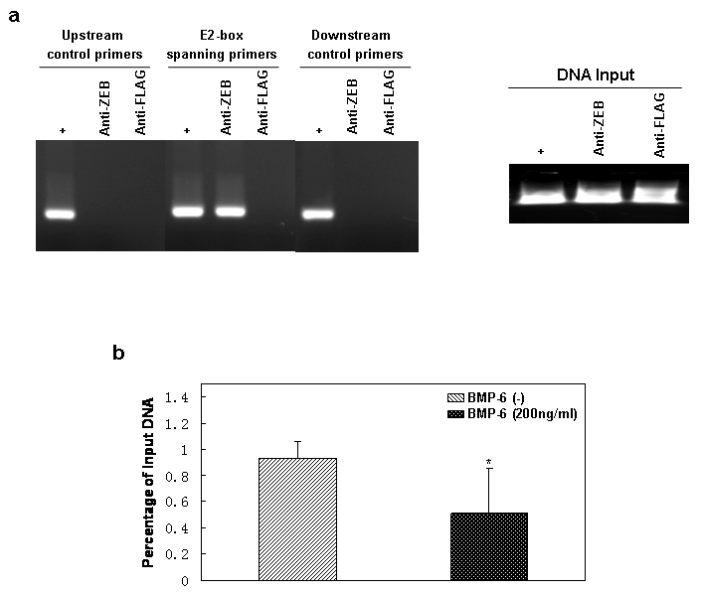
**BMP-6 represses binding of endogenous δEF1 to the E-cadherin proximal promoter in MDA-MB-231 breast cancer cells**. (a) Association of δEF1 with the E-cadherin promoter region was examined *in vivo *by CHIP analysis in MDA-MB-231 cells, using an unrelated anti-FLAG antibody or an anti-ZEB antibody directed against the N-terminal epitopes of δEF1. The amplified human E-cadherin promoter fragment is shown (-175/+21). (b) For quantitative CHIP assay, MDA-MB-231 cells were treated with or without 200 ng/ml rhBMP-6. Cell lysates were collected after 72 h. The IP was performed using anti-ZEB antibody (10 μg) with anti-FLAG antibody (10 μg) as a negative control. DNA fragments containing the E-cadherin promoter region (-175/+21) were amplified by quantitative PCR from anti-ZEB and anti-FLAG immunoprecipitated samples. Data represent three independent experiments. * indicates p < 0.05 in unpaired t-test when compared with un-treated group.

As previously demonstrated, we have shown that BMP-6 can down-regulate the repressor, δEF1, to effectively stimulate the E-cadherin promoter. Considering that δEF1 is directly associated with the E-cadherin proximal promoter *in vivo*, acting as a direct transcription repressor [36], we wished to study this physical interaction in response to BMP-6 by quantitative CHIP assay. Total protein lysates from MDA-MB-231 cells treated with or without 200 ng/ml rhBMP-6 were further incubated with a δEF1 antibody directed against the δEF1 amino-terminus in order to precipitate endogenous δEF1. As shown in Figure 8a, δEF1 specifically bound to the -175/+21 region of the E-cadherin promoter, and the binding was significantly reduced by BMP-6 treatment (Figure 8b). This data supports the notion that BMP-6 stimulation of E-cadherin promoter takes place through both a reduction of δEF1 expression and a physical removal of δEF1 from binding to its cognate DNA elements.

## Discussion

The progressive and metastatic nature of breast cancer has been well recognized, yet the mechanisms through which breast cancer cells acquire their invasive properties have not been clearly elucidated. Several studies have reported that breast cancer produces a variety of growth factors that can play a role in the progression and metastasis of breast cancer. In the current study, we have uncovered that one of these factors, BMP-6, contributes to the regulation of E-cadherin-mediated epithelial-mesenchymal transition of breast cancer *in vitro*. In addition, we have shown that the stimulatory effect of BMP-6 on E-cadherin transcription occurs through reducing the expression and activity of δEF1, which we have found to be a strong transcriptional repressor of the E-cadherin gene. Importantly, the reverse relationship between BMP-6/E-cadherin and δEF1 expressions in cancer cell lines has been verified in clinical tumor specimens. Our results provide the first evidence, at the cellular level, to support the hypothesis that breast cancers may progress and metastasize through the regulation of E-cadherin expression by BMP-6 and δEF1.

Expression of BMPs has been reported to increase with breast cancer progression. Several studies on the overexpression of BMPs, such as BMP-2, BMP-4, and BMP-7 in mammary tumor cells [45-47] are suggestive of a role of BMPs in breast cancer development. Clement *et al*. were the first to report that BMP-6 is detectable, not only in breast cancer cell lines, such as MCF-7, SK-BR-3, MDA-MB-453, BT-20, and ZR-75-1, but also in most tumor specimens, using RT-PCR and immunohistochemistry [21]. Importantly, BMP-6 expression was significantly increased and was most intense in the vicinity of chondroid matrix of complex adenomas and mixed benign tumors of canine mammary glands [1,2]. In agreement with these reports, we present here our finding that significantly higher levels of BMP-6 were observed in breast cancer cell lines and clinical tumor specimens, using quantitative RT-PCR. Moreover, BMP-6 expression is higher in the ER^+ ^breast cancer cell line, MCF-7, compared to the ER^- ^breast cancer cell line, MDA-MB-231. Although BMP-6 expression levels varied widely among tumor specimens, it was relatively higher in ER^+ ^cases than in ER^- ^cases. This coincides with our previous finding that BMP-6 promoter methylation status is correlated with ER status in breast cancer. In that study, we observed significantly lower levels of BMP-6 mRNA in ER^- ^breast cancer cells compared with ER^+ ^breast cancer cells, an effect attributed to hypermethylation status in the ER^- ^breast cancer cells [48]. In addition to breast cancer, BMP-6 has been found in a variety of other cancer cell types, including prostate, kidney, esophagus, and osteosarcoma [25,49-51], suggesting its association with the progression of tumorigenesis.

E-cadherin is ubiquitously expressed by epithelial cells, from which most cancers are derived. E-cadherin-mediated cell-cell adhesion prevents cells in a primary tumor from breaking away and invading near or distant sites. It has been well documented that loss of E-cadherin in mammary epithelial cells can promote breast cancer progression and metastasis [52]. Recently, several factors, including ErbB2 [18], TGF-β [19], and estrogen [20], were reported to regulate E-cadherin expression through different mechanisms. In this study, we have determined that BMP-6 is a novel stimulus of E-cadherin expression in breast cancer cells, providing evidence for a potential role of BMP-6 in tumor progression and metastasis. In addition, our results indicate that BMP-6-induced expression of E-cadherin is correlated with ER status. Higher levels of BMP-6 and E-cadherin transcripts were observed in ER^+ ^breast cancer cells, while lower amounts were detected in ER^- ^cells. These observations are in line with a previous finding that loss of E-cadherin expression was associated with the lack of ER expression and a more aggressive phenotype of breast cancer with poor clinical prognosis [20]. Thus, our observations lead us to propose that BMP-6 may cooperate with other factors, such as estrogen, to participate in attenuating breast caner progression.

Several epigenetic mechanisms have been implicated in E-cadherin regulation in breast cancer [53], among these, a few zinc-finger transcription factors are known to bind to E-box elements of the E-cadherin promoter and repress transcription, including Snail1 [11-14], Snail2 [15,16], and SIP1 [17]. δEF1, a close homolog of SIP1 [54], was previously shown to directly bind to E-box elements of the E-cadherin promoter and inhibit the expression of endogenous E-cadherin mRNA and protein in mammary epithelial cells [36]. These previous observations are consistent with our results demonstrating that overexpression of δEF1 is sufficient to block transactivation of E-cadherin in MDA-MB-231 cells. In line with our findings, a recent paper has also supported a role of δEF1 in E-cadherin repression in lung cancer cells [38]. In spite of these findings, the upstream factors responsible for regulating the δEF1/E-cadherin loop in breast cancer have not yet been identified. Our current work has provided two novel findings in this context, (1) δEF1 can repress BMP-6-mediated up-regulation of E-cadherin and (2) the integrity of a single δEF1-binding element in the proximal E-cadherin promoter is sufficient for the repression effect of δEF1, providing evidence that BMP-6 may affect E-cadherin-mediated progression and metastasis through the regulation of specific genes, such as δEF1, during breast tumorigenesis. Furthermore, the fact that artificial removal of δEF1 by RNAi enhances gene expression of E-cadherin supports the important role of δEF1 to be down-regulated by BMP-6 in the native state in order for BMP-6 to induce E-cadherin expression. RNAi does it by mediating mRNA degradation, whereas BMP-6 does it by (1) inhibiting the expression of δEF1, and (2) by dislodging δEF1 that has already bound to the E-cadherin gene. Our overall data demonstrates that δEF1 is a native repressor of E-cadherin gene. Factors that remove/decrease δEF1 level would subsequently increase the expression of E-cadherin.

Previous research from our group indicated that δEF1 represses BMP-2-induced differentiation of C2C12 myoblasts into the osteoblast lineage, an effect that is not mediated via the canonical BMP/Smad signaling pathway, but instead by differential regulation of the AP-1 pathway [55]. In the study reported here, we found that overexpression of R-Smads (Smad 1 and 5) failed to augment BMP-6-induced transactivation of E-cadherin, which is mediated by suppression of δEF1 (data not shown). However, further studies will be required for a better understanding of the signal transduction mechanisms regulated by BMP-6 and δEF1 in breast cancer cells.

Breast cancer metastasis is a complicated process, during which the important functions of estrogen and the estrogen receptor have been consistently recognized [56,57]. In this study, we observed that, in breast cancer cell lines and clinical tumor specimens, δEF1 was expressed at a higher level in ER^- ^cells compared to ER^+ ^cells, indicating that δEF1 might contribute to the malignant conversion of breast cancer cells towards invasive and metastatic phenotypes. On the other hand, the contribution of BMP-6 to tumor metastasis has been recently studied in prostate cancer, suggesting that BMP-6 plays an important role in regulating tumor cell invasion [25]. We recently showed that BMP-6 in ER^+ ^breast cancer cells could be activated by estrogen through promoter demethylation [29,48]. Considering our finding that BMP-6-induced E-cadherin transactivation occurs indirectly, through the reduction of δEF1 expression, we speculate that overexpression of BMP-6 by breast cancer cells may represent a novel mechanism that regulates specific target genes such as E-cadherin and δEF1, in modulating metastasis and invasion of breast cancer.

## Conclusion

Our data suggest that the stimulatory effect of BMP-6 on E-cadherin transcription in breast cancer cells occurs indirectly, through the reduced expression and activity of δEF1. Repressors of BMP-6 and E-cadherin, such as δEF1, may regulate breast tumor progression and metastasis at different stages, including the initial de-differentiation of primary tumor cells and the maintenance of migratory and/or undifferentiated phenotypes. Further studies should be focused on better understanding of the signal transduction mechanisms and the relationship between BMP-6 and δEF1 in breast cancer formation, progression, and metastasis.

## Competing interests

The author(s) declare that they have no competing interests.

## Authors' contributions

SY conceived the study, carried out the cell culture, luciferase assay, and Q-CHIP assay experiments, and drafted the manuscript. JD performed total RNA extraction and Q-PCR experiments. ZW performed all plasmid construction experiments. WY performed immunoblotting experiments. YQ performed all statistical analyses. MZ and JZ performed RT-PCR experiments. SG, JY, and BS processed the clinical breast tumor samples. TZ supervised the work, and helped to draft and revise the manuscript. All authors have read and approved the final manuscript.

## Pre-publication history

The pre-publication history for this paper can be accessed here:


